# Stable Dietary Ora-Curcumin Formulation Protects from Experimental Colitis and Colorectal Cancer

**DOI:** 10.3390/cells13110957

**Published:** 2024-06-01

**Authors:** Chaitanya K. Valiveti, Balawant Kumar, Anuj D. Singh, Sham K. Biradar, Rizwan Ahmad, Amar B. Singh, Hemachand Tummala

**Affiliations:** 1 Department of Pharmaceutical Sciences, College of Pharmacy & Allied Health Professions, South Dakota State University, Brookings, SD 57007, USA; ckvaliveti@gmail.com (C.K.V.); sham.biradar@sdstate.edu (S.K.B.); 2 Department of Biochemistry and Molecular Biology, University of Nebraska Medical Center, Omaha, NE 68198, USA; balawant.kumar@unmc.edu (B.K.); anuj.singh4884@gmail.com (A.D.S.); rizwan.ahmad@unmc.edu (R.A.); 3 Veterans Affairs Nebraska—Western Iowa Health Care System, Omaha, NE 68105, USA

**Keywords:** IBD, CRC, curcumin, dietary products, Ora-curcumin, polymer–drug complexes

## Abstract

Inflammatory bowel disease (IBD) is a chronic gut disorder that also elevates the risk of colorectal cancer (CRC). The global incidence and severity of IBD are rising, yet existing therapies often lead to severe side effects. Curcumin offers potent anti-inflammatory and chemotherapeutic properties. However, its clinical translation is hindered by rapid metabolism, as well as poor water solubility and stability, which limits its bioavailability. To address these challenges, we developed OC-S, a water-soluble and colon-targeted curcumin formulation that protects against colitis in mice. The current study advances OC-S as a dietary supplement by establishing its stability and compatibility with various commercial dietary products. Further, OC-S exhibited specific binding to inflamed colon tissue, potentially aiding in targeted drug retention at the inflammation site in colitis with diarrhea symptoms. We further investigated its efficacy in vivo and in vitro using a murine model of colitis and tumoroids from APC^min^ mice. OC-S significantly reduced colitis severity and pro-inflammatory cytokine expression compared with curcumin, even at very low doses (5 mg/kg/day). It also demonstrated higher anti-proliferative activity in CRC cells and colon cancer tumoroids vs. curcumin. Overall, this study demonstrated that OC-S effectively targets and retains water-soluble curcumin at the inflamed colon sites, while showing promise in addressing both colitis and colorectal cancer, which potentially paves the way for OC-S to advance into clinical development as a dietary product for both IBD and CRC.

## 1. Introduction

Inflammatory bowel disease (IBD) is a chronic autoimmune disorder of the intestine comprising primarily ulcerative colitis (UC) and Crohn’s disease (CD). The prevalence of IBD is increasing worldwide and carries enormous burdens on the healthcare system, including a higher risk of developing colorectal cancer compared with the general population. Unfortunately, despite the recent advances in biologics to manage IBD, disease severity and risk of colon cancer have not improved significantly, which leads to colectomy and poor quality of life. Notably, colitis-associated colon cancer (CAC) is more aggressive than spontaneous colon cancer (CRC), and the risk of colon cancer in young adults is increasing [1,2,3,4,5]. Taken together, new approaches are needed for the improved management of IBD and colon cancer. The current research in developing therapeutics against IBD and colon cancer risk is focused on identifying/developing novel compounds and/or formulations with minimal side effects and bioavailability to induce and/or maintain remission without major side effects. Thus, it is imperative that in addition to the pursuit of “safe precision medicine”, we develop and test complementary and alternative medicines (CAMs) to effectively treat and/or manage IBD and colon cancer [6].

Curcumin, a polyphenol, is a popular food additive and a traditional Ayurvedic medicine. Furthermore, curcumin has been shown to have the potential as a preventive and therapeutic agent for IBD and CRC, in preclinical studies [7,8,9,10,11,12]. Despite its demonstrated potential, the success of curcumin is yet to be translated into human applications due primarily to its poor bioavailability caused by its extremely low water solubility (~1 µg/mL), and rapid metabolism [13,14,15,16,17]. Also, curcumin is unstable at various pHs and extremely prone to photodegradation [18,19]. Recent studies, including ours, have proved that novel technologies can help improve curcumin bioavailability [20,21,22,23,24,25]. In this regard, we have developed the Ora-Curcumin (referred to as OC-S hereon) technology by non-covalent complexation of curcumin with a distinct group of polymers known as Eudragits^®^. Eudragit^®^ S100 is selected for complexation with curcumin for two reasons. Firstly, it is a pH-sensitive copolymer that remains insoluble in acidic environments but becomes soluble when the pH exceeds 6.8. Due to this property, it has been used for decades as a coating material for colon-targeted drug delivery systems. However, in this manuscript, it is used as a complexing agent to enhance the solubility of curcumin and achieve its colon-targeted delivery. Secondly, pathological changes during mucosal inflammation involve the depletion of the mucus layer and the accumulation of positively charged proteins like transferrin, anti-microbial peptides, and eosinophil cationic proteins. Given the positive charge of the inflamed site surface, we hypothesize that the negatively charged surface of Eudragit^®^ S100 will adhere to the positively charged inflamed surface, allowing it to retain the drug for a longer duration and effectively release it.

We have further demonstrated that OC-S has remarkably high solubility, exceeding that of curcumin by more than 2000 times, and it can be engineered to deliver soluble and bioactive curcumin to the colon lumen, offering protective benefits against experimental colitis in mice [24]. However, the goal of the current study is to advance OC-S further as part of a diet to achieve its therapeutic benefits in preventing inflammation in colitis and reducing the risk of colorectal cancer. For incorporation into dietary regimens, OC-S must demonstrate stability and retention of bioactivity with food products and require significantly lower effective doses compared with its therapeutic dosage, as a drug shown in the previous study.

Accordingly, in the current study, we investigated the compatibility, stability, and retention of the bioactivity of OC-S within multiple commercial dietary products, as well as its efficacy in exhibiting anti-inflammatory and anti-neoplastic effects through in vitro and ex vivo models. Moreover, we tested the therapeutic efficacy of OC-S at very low doses (curcumin equivalent of 5 mg/kg/day) on mice subjected to experimental colitis. Taken together, this study signifies the potential of integrating OC-S as a dietary supplement for therapeutic gain in people suffering from gastrointestinal inflammation, including IBD, and as an adjuvant therapy for colon cancer.

## 2. Material and Methods

### 2.1. Materials

The Eudragit^®^ S100 polymer was provided by Evonik Industries (Birmingham, AL, USA). Curcumin from TCI (> 98% pure; Portland, OR, USA) and solvents of HPLC grade (acetone, dimethyl sulfoxide (DMSO), methanol, ethanol) were purchased from Fisher Scientific (Pittsburgh, PA, USA). Polyvinyl alcohol was purchased from MP Biomedicals (Santa Ana, CA, USA). Dulbecco’s modified Eagle’s medium (DMEM), fetal bovine serum (FBS), penicillin-streptomycin, and trypsin-ethylenediaminetetraacetic acid (EDTA) were purchased from Life Technologies (Carlsbad, CA, USA). Lipopolysaccharide (LPS) from Escherichia coli serotype 055:B5 was purchased from Sigma-Aldrich (St. Louis, MO, USA). Dextran sulfate sodium (DSS) was purchased from TdB Labs, Sweden. Antibodies for Western blot analysis were obtained from Cell Signaling Technology, Inc. (Beverly, MA, USA; Supplementary Table S1). Caco2 and HCT116 cells were either purchased from ATCC (American Type Culture Collection, Rockville, MD, USA) or were available in our laboratory. At the UNMC core facility, cell lines are frequently tested for authenticity by genomic analysis.

### 2.2. Preparation of OC-S Complexes

The OC-S was formulated using the nano-precipitation method described previously [24]. The OC-S formulation was prepared by dissolving the drug and polymer in a 1:3 ratio using an organic solvent mixture (50% acetone in DMSO; 4 mL). This solution was then added to an acidic aqueous solution containing 3% *w*/*v* polyvinyl alcohol as a stabilizer under constant stirring at 600 rpm, allowing for controlled precipitation. The mixture was further stirred for 16 h at room temperature in dark conditions to evaporate the organic solvent, facilitating the non-covalent complexation of curcumin as a yellow precipitate. The resulting dispersion was centrifuged at 20,000× *g* for 30 min to collect the complexes. These were washed three times, resuspended in deionized water, freeze-dried, and stored at −20 °C until use. Each batch of complexes was characterized by FTIR, solubility in 50 mM phosphate buffer, pH 7.2, and formation of complexes as described previously [24]. The loading of curcumin on the polymer was determined as described previously [24].

### 2.3. Preparation of Functional Dietary Products (DP) with OC-S Complexes (OC-S-DP)

Sanford profile^®^ nutritional grade dietary products were chosen for this study. In this study, we selected pomegranate green tea, mixed fruit drink, chicken soup, and orange drink. These are solid powder sachets that are instructed to be mixed with water for daily intake. Freshly prepared OC-S equivalent to 10 mg of pure curcumin (based on drug loading) was mixed with the dietary powders by geometric dilution method before they were sealed back in the original sachets (OC-S-DP) and stored at room temperature under dark conditions.

### 2.4. The Extraction of Curcumin from OC-S-DP

The efficiency of extracting curcumin from OC-S-DP was assessed through the screening of various solvents, including methanol, acetonitrile, ethyl alcohol, isopropyl alcohol, and a combination of acetonitrile and methanol (60:40) To extract curcumin, the organic solvent was added to the dietary product and sonicated for 30 min using a bath sonicator. Subsequently, the product was centrifuged at 20,000× *g* for 30 min and the supernatant was collected. The supernatant was filtered through a 0.2 μm filter and diluted further using the same solvent before injecting it into a reverse-phase High-Performance Liquid Chromatography (RP-HPLC) system (Section 2.5). The area under the curve was used to calculate the amount of curcumin in the extract. Additionally, to determine the extraction efficiency, the dietary products were supplemented with known quantities of pure curcumin, and the extracted curcumin was subsequently quantified through RP-HPLC.

### 2.5. Quantification of Curcumin Content from the OC-S-DP

An assay method was developed to determine the content of curcumin present in the dietary supplement by using RP-HPLC**.** The RP-HPLC analysis was conducted using an isocratic elution method with the Syncronis C18,150 × 4.6 mm, 5 µm column connected to Waters HPLC system (Milford, MA, USA) equipped with a 1525 binary pump and a W2998 PDA detector. The mobile phase contained acetonitrile and 0.05 M citrate buffer pH 4.4 (60:40) and used a flow rate of 1.0 mL/min with a detection wavelength of 425 nm. The injection volume was 20 µL. The area under the curve was used to analyze the unknown sample concentration against the calibration curve range of curcumin from 0.01–0.4 µg/mL. An extracted sample from the original dietary product without the addition of OC-S was used to identify and separate any interference peaks.

### 2.6. Curcumin Stability with OC-S-DP

To conduct stability and compatibility investigations, the sealed sachets containing OC-S-DP were preserved at room temperature in dark conditions for 12 months. At predefined intervals, samples of OC-S-DP were precisely weighed to match 0.2 mg of pure curcumin. The curcumin present in the sample was then extracted using a blend of acetonitrile and methanol (60:40) and processed as per the aforementioned procedure (Section 2.4). The quantity of stable curcumin in the extract was subsequently determined using RP-HPLC (Section 2.5).

### 2.7. Simulated Gastric Extraction of OC-S-DP for Functional Studies

Using the geometric dilution method, OC-S equivalent to 2.0 mg of pure curcumin was mixed with 1.0 g of each dietary product and stored in the dark at room temperature for one month in a sealed container. To this mixture, five mL of 0.1 N hydrochloric acid was added and the solution was incubated for 3 h at 100 RPM and 37 °C. Following incubation, the pH of the samples was adjusted to 7.0 using 1 N sodium hydroxide and diluted to 10.0 mL with 50 mM phosphate buffer and a pH of 7.0. The diluted samples were further incubated for three additional hours. Subsequently, the samples were centrifuged at 20,000× *g* for 30 min the resultant supernatants were collected, filtered through a 0.2 µm membrane, lyophilized, and stored at −20 °C for further functional studies.

### 2.8. Preparation of OC-S and Curcumin Solutions for In Vitro Use

OC-S and curcumin were dispersed in PBS at a curcumin equivalent of 0.5 mg/mL. The mixture was centrifuged at 20,000× *g* for 30 min and the supernatant was sterilized by passing through a 0.2 µm filter. The stock solutions were diluted in PBS. The cells were treated at 10 and 50 µg/mL equivalent concentrations of curcumin. The amount of OC-S used was adjusted to the amount of curcumin present in the formulation (drug loading) for each batch determined as shown previously [24].

### 2.9. CRC Cells and APC^min^ Tumoroids Culture

CRC cells were maintained in Dulbecco’s modified eagle’s medium (DMEM) or an RPMI-1640 medium, as appropriate, which were supplemented with 10% fetal bovine serum (FBS) and penicillin–streptomycin (50 µg/mL). Colonic tumoroid was established from APC^Min^ mice as described in [26]. These tumoroids were embedded in a Matrigel bed in a 24-well culture dish and bathed in a special cell culture medium containing N2 (50 µM) and B27 (100 µM) as supplements, as well as EGF (100 ng/mL).

### 2.10. Western Blot Analysis

Western blot was carried out as described previously [27]. Briefly, cells, tumoroids, or tissue lysates were subjected to sonication before centrifugation at 12000× *g* at 4 °C for 10 min. The amount of protein was determined using the Bradford method (Bio-Rad, Hercules, CA, USA), and samples were prepared using 6× SDS loading dye. Prepared protein samples were resolved using SDS-PAGE, and the signal was visualized with horseradish peroxidase-conjugated secondary antibodies using enhanced chemiluminescence (Amersham Biosciences, Piscataway, NJ, USA # A38555; Supplementary Table S1).

### 2.11. RNA Isolation and Real-Time qPCR Analysis

RNA was extracted from tissue or cells using a Quick-RNA Miniprep Kit (Zymo Research, Irvine, CA, USA). cDNA was prepared using iScript cDNA synthesis kit (Bio-Rad). The real-time qPCR reactions were performed with appropriate primers set using 20 ng of cDNA/reaction and 2×iQTM SYBR Green Supermix (Bio-Rad) (Supplementary Table S2).

### 2.12. Animals and Colitis Induction

Mice used in this study were from a C57BL/6 background. They were kept in individually ventilated cages in compliance with the approved protocol from the University of Nebraska Medical Center, Omaha (protocol # 17-126-11FC). Colitis was induced in mice using Dextran Sodium Sulfate (DSS) as described previously [28]. Briefly, male (*n* = 18) and female (*n* = 6) mice (10–12 weeks old) were provided regular drinking water or 2.5%DSS (*w*/*v*; MW = 36–40 kD, TdB Labs, Sweden) for nine days. For blood absorption studies, on day 7 of DSS treatment, mice were ingested with OC-S (15 mg/kg curcumin equivalent) through oral gavage. After one hour, 30 microliters of blood was collected to determine the levels of absorbed curcumin in the plasma. The 1 h time point for plasma represents the time for the peak plasma concentration (T_max_) in mice for curcumin. The DSS/water consumption was monitored, and mice were weighed daily and visually inspected for loose stool and/or rectal bleeding. Body weight loss was calculated as the percent difference between the starting body weight and the actual body weight on a given day. Animals were sacrificed on day nine, and colons were removed and assessed for colonic thickness or edema, which was determined by the ratio of colon weight to length. Further, Swiss rolls were made to evaluate the histopathological analysis using the following parameters by the UNMC GI-pathologist in a blinded manner: inflammation (score of 0–3), percentage involved in inflammation (score of 1–4), depth of inflammation (score of 0–3), crypt damage (score of 1–4), and percentage involved in crypt damage (score of 1–4). The overall cumulative injury score was calculated using scores from these parameters.

### 2.13. Evaluation of Inflammation-Specific Binding of OC-S Complexes

Mouse (C57BL/6) colons from healthy and DSS (2.5% *w*/*v* for 7 days)-induced colitis were transversely opened and exposed to 1 mg/mL of soluble OC-S (Section 2.8) for 45 min. Post incubation, the colons were rinsed thrice with PBS for 5 min each. Subsequently, the colon segments were subjected to imaging at λ-470/535 nm to detect bound curcumin to the mucosal wall using Bruker In-Vivo imager (Billerica, MA, USA). The relative quantity of curcumin bound was determined by measuring the sum intensities of fluorescence signal obtained from the images using Bruker image processing software.

### 2.14. Statistical Analysis

Statistical analysis was performed using Student’s *t*-test or one-way ANOVA and corrections for multiple comparisons were made using Tukey’s multiple comparison tests in GraphPad Prism 10.1.0 (GraphPad Software, Inc., Boston, MA, USA). A *p*-value < 0.05 was defined as statistically significant. All data presented are representative of at least three repeat experiments and are presented as mean ± SD.

## 3. Results

### 3.1. OC-S Is an “Inflammation Site-Targeted Local Drug Delivery System”

The OC-S complexes were prepared by the nanoprecipitation of Eudragit S100^®^ and curcumin and characterized for the complex formation and pH-dependent solubility, as described previously [24]. The ability of OC-S to deliver soluble curcumin to the colon of healthy mice was established previously [24]. However, considering the observation that intestinal permeability is altered in IBD patients, absorption studies were also conducted in mice subjected to colitis, developed by exposure to 2.5% (*w*/*v*) of Dextran Sulphate Sodium (DSS) in drinking water. Similar to healthy mice [24], no detectable curcumin was observed in the plasma of colitis mice 1 h post oral administration of OC-S (15 mg/kg/day) (*n* = 8). One timepoint was considered, as it represents the time for peak plasma concentration (T_max_) for curcumin as shown in our previous studies in mice [19]. Together, the above data estrablishd the potential of OC-S to selectively deliver soluble curcumin to the lumen of the colon without exposing it to systemic circulation in both healthy and colitis mice.

**Figure 1:**
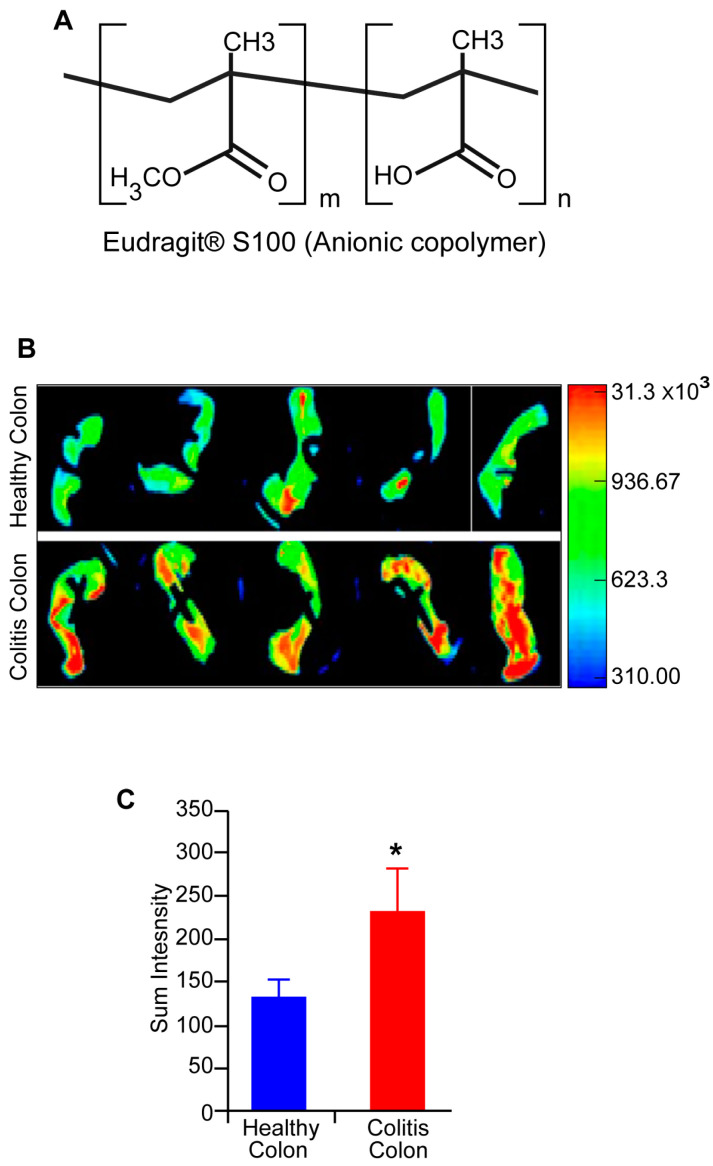
Increased binding of OC-S to the injured and inflamed colon of mice subjected to DSS colitis: OC-S complexes, formulated with negatively charged Eudragit^®^ S100 (**A**), at a concentration of 1 mg/mL, were incubated with either healthy or inflamed colon tissue. The binding of OC-S to the colon mucosa was assessed through imaging at λ-470/535 nm using Bruker In-Vivo Xtreme molecular imaging software (v. Rev. B12/12). The figure further displays a representative image of curcumin fluorescence; (**B**) and the corresponding sum of fluorescence intensities determined using Bruker software, and (**C**). Values are presented as a mean ± SD. * *p* < 0.05.

Additionally, OC-S is engineered to carry a negative charge at the colon pH through the selection of an anionic polymer, Eudragit^®^ S100 (Figure 1A), which may provide an additional benefit of charge-based interactions with the positively charged inflamed tissue [29,30,31]. Indeed, when transversely opened colons from control and colitis-challenged mice were incubated with OC-S, a significantly higher binding of OC-S to the inflamed colon tissues was observed compared with healthy colon tissues (Figure 1B,C). Quantitative analysis of the differential binding, based on fluorescent imaging at curcumin fluorescence wavelength at λ-470/535, further revealed that approximately 75% more OC-S was bound to inflamed colitis tissues than healthy colon tissues (Figure 1B), Overall, these findings validated the efficacy of OC-S not only target the delivery of soluble curcumin locally to the lumen of the colon but also to provides specific binding to the inflamed colon tissue.

### 3.2. OC-S Attenuates Dextran-Sulfate-Sodium-Induced Mouse Colitis at a Very Low Dose

**Figure 2:**
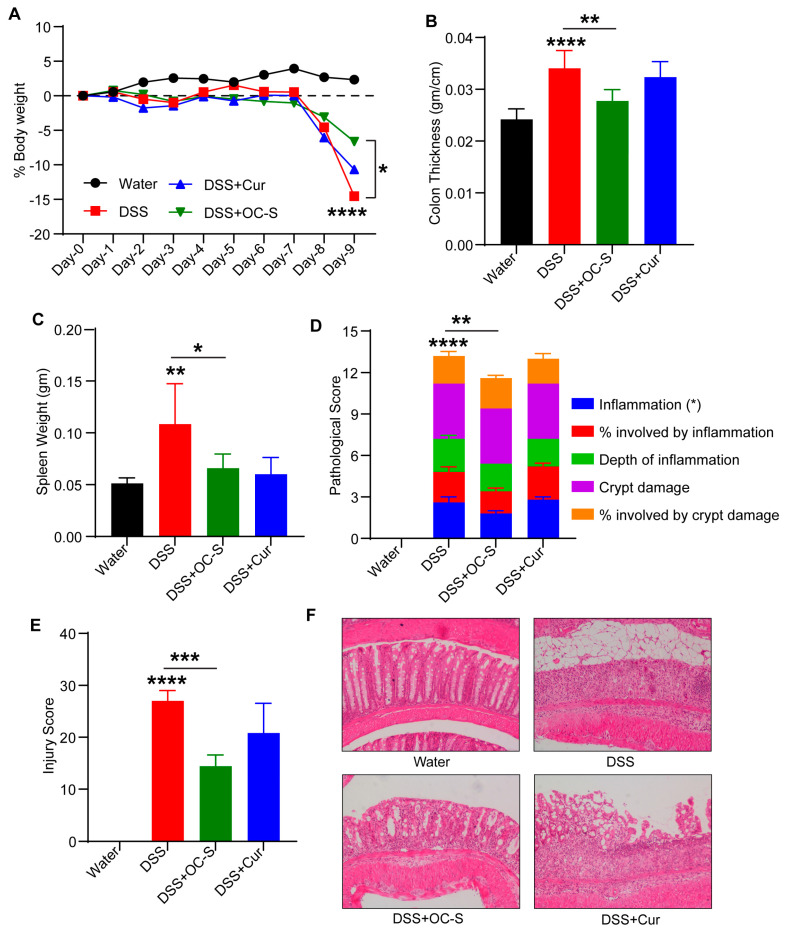
OC-S promotes mucosal healing in murine models of colitis. The colitis was induced in C57BL6 mice by administration of 2.5% DSS (*w*/*v*) in the drinking water for 9 consecutive days. DSS-subjected mice were treated with curcumin (5 mg/kg body weight) and OC-S (5 mg/kg body weight): (**A**) percent decrease in body weight during 9 days for DSS alone, DSS+ curcumin, and DSS + OC-S treatment, respectively; (**B**) colon thickness (edema) evaluated as weight (gm)/cm decreases with OC-S treatment compared with DSS; (**C**) DSS + OC-S treatment reduces the spleen weight versus DSS treated mice; (**D**,**E**) OC-S treatment group mice show reduced inflammation and associated injury compared with DSS-treated alone; and (**F**) representative H&E images of the colonic tissues from control, DSS-treated, DSS+ curcumin, and DSS + OC-S treatment group mice. Values are presented as a mean ± SD. * *p* < 0.05, ** *p* < 0.01, *** *p* < 0.001, **** *p* < 0.0001.

Given that OC-S specifically targeted the inflamed tissue in the colon, as a part of a larger goal of delivering it as a dietary supplement, our next objective was to examine the possible lowest dose of OC-S for inhibiting colitis in a preclinical animal model. In the current study, we compared the efficacy of OC-S with unformulated curcumin at a dose of 5 mg/kg/day given orally in two divided doses. Mice subjected to DSS colitis, as described in methods, were monitored for changes in body weight, loose stools/diarrhea, or rectal bleeding and diarrhea. As Shown in Figure 2A–C, the oral administration of DSS induced significant body weight loss, thickening of the colon, and an increase in spleen weight compared with control mice. OC-S administration even at a dose of 5 mg/kg mitigated colitis-associated weight loss, colon thickening, and spleen weight (Figure 2A–C). We did not observe substantial changes in these parameters when mice were subjected to DSS along with curcumin. Histopathological examination of H&E colon sections indicated an acute inflammatory response with mucosal erosion, congestion, edema, reduction in crypts, and infiltration of inflammatory cells in the DSS group (Figure 2D–F). Compared with the DSS colitis and curcumin groups, mucosal inflammatory cell infiltration, erosion, and edema in the OC-S-treated group were significantly improved (Figure 2D–F). Overall, our data suggested that compared with curcumin, OC-S treatment even at a very low dose of 5 mg/kg/day was effective against experimental colitis.

### 3.3. OC-S Treatment Re-Equilibrates Inflammatory Cytokines to Reduce Colonic Inflammation

**Figure 3:**
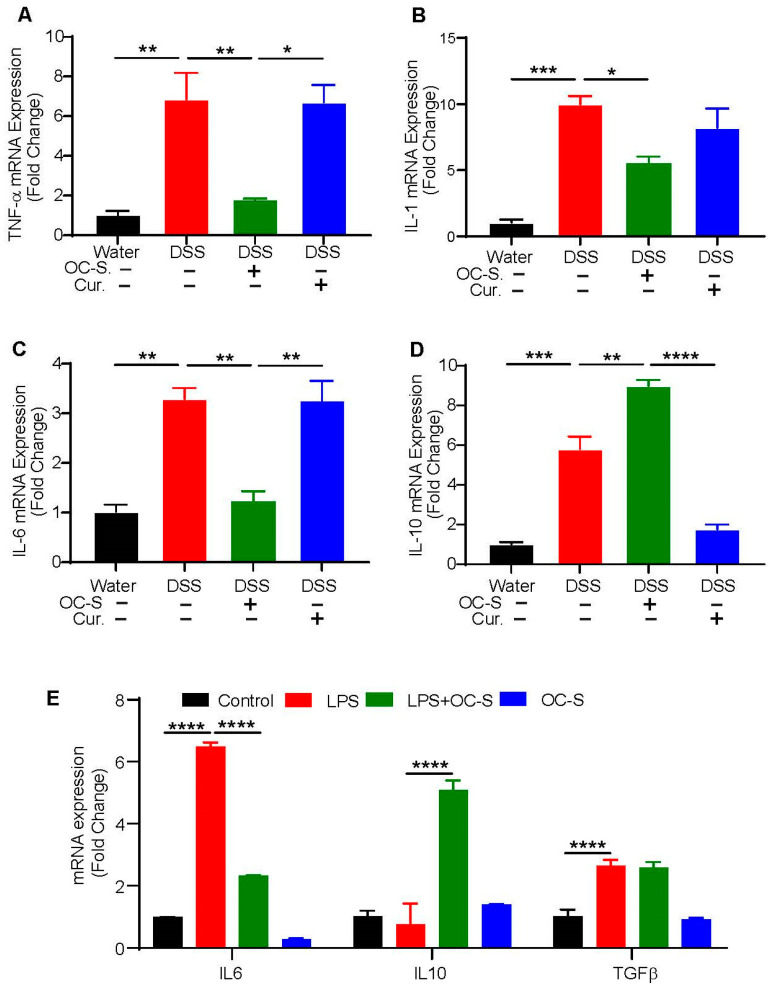
OC-S reduces the pro-inflammatory signaling and increases anti-inflammatory cytokine in the colonic mucosa. Total RNA was subjected to qRT-PCR to examine the expression of pro-inflammatory and anti-inflammatory cytokines: (**A**–**D**) OC-S treatment reduces the level of TNF-α, IL-1, and IL-6 in mice colons while increasing the anti-inflammatory cytokine (IL-10); (**E**) modulation of expression of IL-6, Il-10, and TGFβ in epithelial cells exposed to LPS (2 µg/mL) and OC-S alone or in combination. Values are presented as a mean ± error. * *p* < 0.05, ** *p* < 0.01, *** *p* < 0.001, **** *p* < 0.0001.

Cytokines are the critical pathophysiological factors that regulate colonic inflammation initiation, progression, and resolution [32,33,34]. Therefore, we next examined the level of cytokines like TNF-α, IL-1, IL-6, and IL-10 in the colonic tissue of mice subjected to DSS and in the DSS mice that were treated with OC-S and curcumin. As shown in Figure 3A–D, the TNF-α, IL-1, IL-6, and IL-10 levels in the DSS group were markedly higher than in control mice. In comparison, TNF-α, IL-1, and IL-6 levels were significantly reduced in the OC-S treatment group compared with curcumin (Figure 3A–C). In contrast, IL-10 levels in OC-S-treated mice were higher compared with the DSS- and curcumin-treated mice (Figure 3D). Overall, our data suggested that OC-S recalibrated the colitis-associated gut inflammatory milieu potentially by regulating the expression of inflammatory cytokines.

### 3.4. OC-S Modulates LPS-Induced Inflammatory Responses in Intestinal Epithelial Cells

To further explore the effect of OC-S in the regulation of inflammatory processes in epithelial-intrinsic manners, we subjected IECs to LPS treatment (2 µg/mL) to simulate the inflammatory processes with and without OC-S. The impact on key inflammatory cytokines IL-6, IL-10, and TGFβ was analyzed using qRT-PCR. As shown in Figure 3E, LPS treatment induced a significant increase in the expression of IL-6 and TGFβ (Figure 3E). Expression of IL-10 expression was not significantly altered (Figure 3E). Consistent with its anti-inflammatory properties, OC-S attenuated the LPS-induced decrease in IL-6 expression compared with the curcumin pretreatment (Figure 3E). In contrast, IL-10 expression was significantly upregulated in OC-S + LPS-treated Caco-2 cells (Figure 3E). The OC-S treatment had no major effect on the LPS-induced TGFβ expression. Collectively, these findings suggested an immunomodulatory role for OC-S in specifically altering IL-6 and IL-10 expressions.

### 3.5. OC-S Improved Therapeutic Efficacy of Curcumin in CRC Cells and Colon Tumoroids

**Figure 4:**
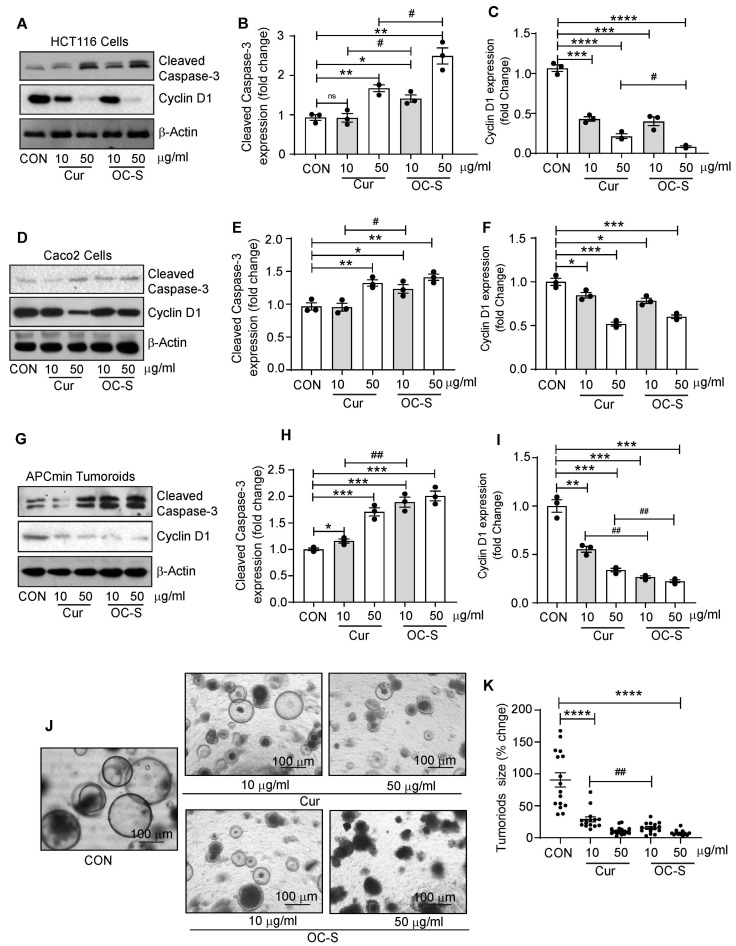
The OC-S treatment induces cell death over curcumin, in CRC cells and APCmin tumoroids: (**A**–**C**) representative immunoblotting and densitometry of cleaved caspase-3 and cyclin D1 in HCT116 cells treated with curcumin and OC-S in a dose-dependent manner 10 and 50 µg/mL; (**D**–**F**) representative cleaved caspase-3 and cyclin D1 immunoblot and its densitometry analysis from whole-cell lysate of Caco2 cell treated with curcumin and OC-S; (**G**–**I**) cleaved caspase-3 and cyclin D1 expression and their densitometry from APCmin-derived cell lysate treated with curcumin and OC-S in a dose-dependent manner, (**J**,**K**) phase-contrast images of APCmin tumoroids treated with curcumin and OC-S for 24 h; tumor size was analyzed by Image J software (v. 1.53K). Values are mean ± SD. * *p* < 0.05, ** *p* < 0.01, *** *p* < 0.001 **** *p* < 0.0001, # < 0.05, ## < 0.01. ns: not significantly.

Given the established association between inflammation and colon cancer, it is reasonable to infer that curcumin’s potent anti-inflammatory properties should also translate into anti-colon-cancer effects. Thus, to evaluate the efficacy of OC-S over curcumin, we analyzed the expression of Cyclin D1, a proliferative marker, and Cleaved caspase-3, an apoptotic marker, respectively, in CRC cells. For this, HCT116 cells were treated in a dose-dependent manner, and whole-cell lysate was used for the analysis of Cyclin D1 and Cleaved caspase-3. Immunoblotting and densitometry analysis demonstrated that OC-S treatment significantly inhibited cyclin D1 expression compared with curcumin treatment in a dose-dependent manner (Figure 4A–C). In contrast, cleaved caspase-3 expression was significantly upregulated in OC-S-treated CRC cells compared with the curcumin-treated CRC cells. To ascertain that these effects are not cell-specific, we further treated the Caco-2 cell with OC-S and/or curcumin. OC-S treatment resulted in a similar outcome (Figure 4D–F). We further determined the efficacy of OC-S using EX-vivo tumoroid culture. Twenty-four hours post treatment, phase-contrast images were captured to determine the effects on tumoroid growth. These tumoroids were then collected for immunoblotting. As shown in Figure 4G–I, immunoblotting and densitometry analysis of Cyclin D1 and Cleaved caspase-3 exhibits outcomes similar to the treatment of CRC cells. Tumoroid size was also reduced significantly in OC-S in a dose-dependent manner in contrast to the curcumin-treated tumoroids (Figure 4J,K). Taken together, our data suggested a role of OC-S in inhibiting colon carcinogenesis than curcumin.

### 3.6. Compatibility and Stability of OC-S in Dietary Products as a Supplement

Once we established that OC-S effectively delivers soluble curcumin to the colon lumen and exerts protective effects against colon inflammation and CRC cell growth, the next logical question was if OC-S can be delivered bio-stably as a dietary supplement to manage the disease flare in IBD patients and to reduce the risk of colon cancer. To test this, OC-S was integrated into multiple commercially available dietary products (including pomegranate green tea, mixed fruit drink, chicken soup, and orange drink), packaged in single-use sachets, resulting in OC-S-DPs. Two primary challenges in this approach were identified: (a) the potential incompatibility of OC-S with dietary products due to the inherent instability of curcumin and (b) the potential interference of dietary components with the availability and pharmacological function of OC-S.

**Figure 5:**
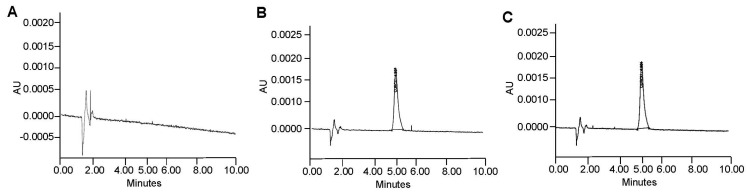
RP-HPLC method to quantify pure curcumin and curcumin from OC-S: The solvent used for sample preparation did not show any peaks (**A**), while pure curcumin (**B**) and curcumin extracted from OC-S (**C**) showed a distinct peak at a retention time of around 5 min.

**Figure 6:**
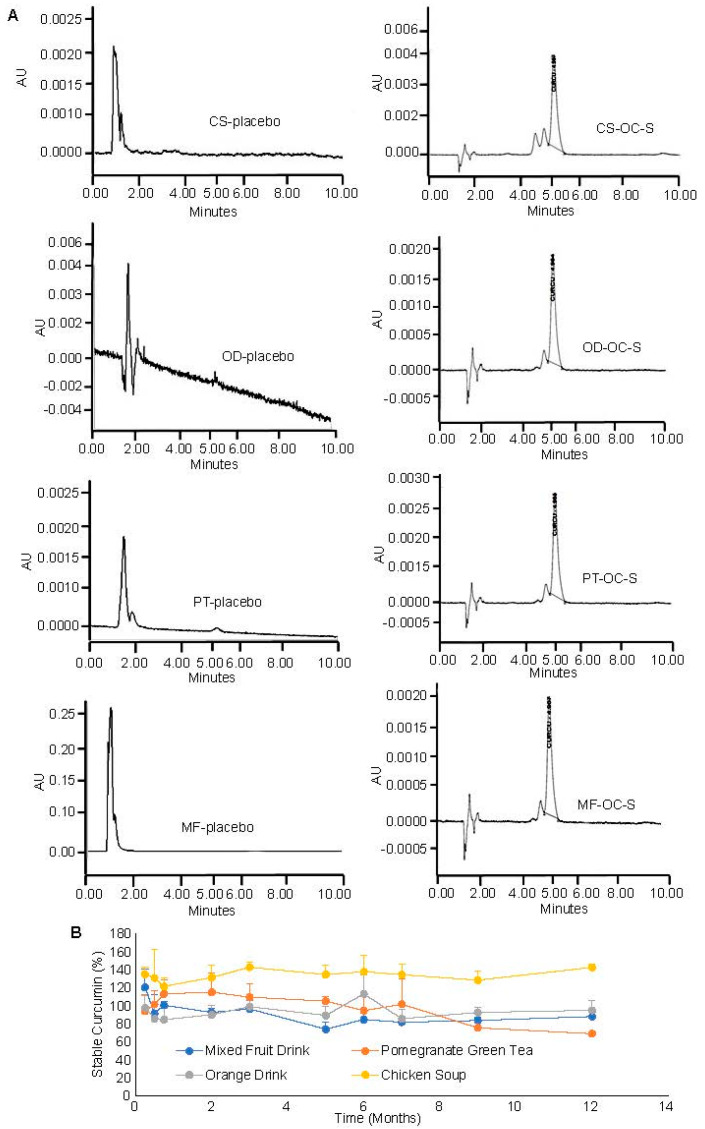
Extraction and HPLC-based detection method for quantifying stable curcumin present in the OC-S-DPs: (**A**) There is a distinct curcumin peak observed from OC-S-DPs extracts at around 5 min retention time (right panel) that is absent when OC-S is not added to the DPs (left panel), indicating the absence of interfering substances from DPs, and (**B**) the stability of curcumin in the dietary supplements was assessed over a 12-month period using the aforementioned method, which indicates that OC-S complexes were stable in dietary supplements.

Before investigating the stability and compatibility of OC-S with DPs, specific extraction and detection (by RP-HPLC) methods were established to quantify the amount of stable curcumin present in OC-S-DPs. The methods were optimized to mitigate interference from dietary components and other excipients from OC-S formulations during analysis (Figure 5 and Figure 6). Various organic solvents such as methanol, acetonitrile, ethyl alcohol, isopropyl alcohol, and acetonitrile:methanol (60:40) were assessed for extracting curcumin from OC-S-DPs. The combination of acetonitrile and methanol (60:40) exhibited the highest extraction efficiency with minimal interfering substances from multiple OC-S-DPs. The extracted sample containing curcumin was then injected into an HPLC column to separate interfering substances, as outlined in the Methods Section 2.4. The absence of interfering substances from the extract for the quantification of curcumin was confirmed by the lack of HPLC peak at curcumin retention time (~5.3 min) from the extracts of dietary products without the addition of OC-S (placebo) (Figure 6A, left panel). HPLC peaks representing the extracted curcumin from OC-S-supplemented dietary products are shown in Figure 6A in the right panel. The extraction efficiency, reported as mean percent extraction ± SD (*n* = 3), varied among the functional dietary products (80–100%) depending on the product and batch.

The chemical and functional stability of OC-S was carefully examined during a 12-month storage period in darkness at room temperature when combined with the dietary products. At specific intervals, sealed sachets were opened, and the amount of stable curcumin present was determined as mentioned in the above paragraph. The CS-OC-S sample showed consistent curcumin content from 0 to 12 months of values 96.9± 15.6 to 105.9 ± 1.8, respectively. Later, we observed that the chicken soup had spice condiments which may contain turmeric powder (curcumin) in the sliced chicken pieces, which might have contributed to the detection of more than 100% of added curcumin in the sample extraction. These amplified peaks in the chromatogram were specifically observed when chicken pieces were included in the sample and therefore impacted the standard deviations in a few samples. On the other hand, PT-OC-S showed a trend of a decrease in curcumin content range from 108.1 ± 7.8 to 73.6± 0.7. The rest of the functional dietary supplements hold the curcumin integrity from degradation throughout the 12 months (Figure 6B). There is one important observation: the standard deviations are on the higher side for the solid stability samples. This could be due to OC-S complexes partitioning or physically adsorbing onto the surrounding matrix of dietary supplements. This might cause the sampling errors. The data are reported as mean ± SD with *n* = 3.

### 3.7. OC-S Food Extract Shows Therapeutic Efficacy in Colon Cancer Cells

**Figure 7:**
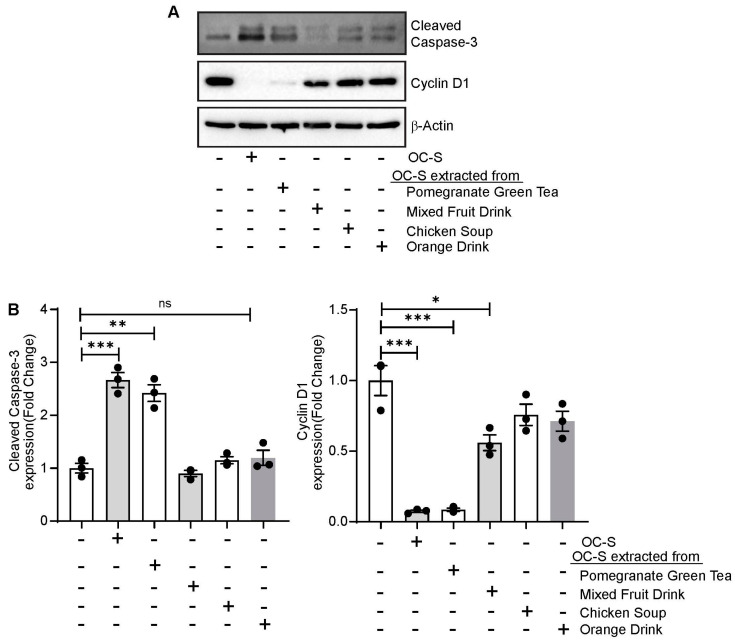
OC-S extracted from dietary food products induces apoptosis and inhibits cell proliferation in colon cancer cells: (**A**,**B**) Representative immunoblotting and densitometry analysis of Cleaved caspase-3 and Cyclin D1 in Caco2 cells treated with different food extracts. Values are mean ± SD. (*** *p* < 0.0001, ** *p* < 0.001, * *p* < 0.05). ns: not significantly.

To simulate in vivo conditions, after one month of incubation together, the OC-S-DPs were treated with 0.1 N HCl for 3 h followed by adjusting the pH to 7.0 for a further 3 h incubation in PBS at pH 7.0. As only a soluble part of the drug is biologically active, the soluble portion of this mixture was subsequently isolated by centrifugation and filtration. There was no sonication used in this experimental setup to facilitate the solubility of curcumin or OC-S to simulate in vivo conditions. Pure OC-S was used as a positive control. Caco2 cells were treated for 24 h. Cells were harvested post treatment, and total-cell lysate was used for immunoblotting. As shown in Figure 7A,B, immunoblotting and densitometry analysis show significant upregulation of Cleaved caspase-3 in OC-S-treated cells. OC-S from pomegranate green tea and mixed fruit drink extract show significant upregulation of cleaved caspase-3 and downregulation of cyclin D1 compared with control cells, respectively. OC-S extract from chicken soup and orange drink showed no significant difference in Cyclin D1 or Cleaved caspase-3 compared with control cells (Figure 7A,B). Taken together, our results suggest that the soluble portion of OC-S is not only stable in food extracts but also biologically active for anti-proliferative potential.

## 4. Discussion

This current research focuses on preparing and testing palliative therapeutic agents that can be used to mitigate disease symptoms and/or progression without major side effects. While curcumin exhibits anti-inflammatory and anti-tumor activities, one of the major challenges associated with curcumin’s use as a therapeutic agent is its relatively low bioavailability [7,35,36,37,38,39,40,41]. Curcumin exhibits low solubility and stability in water, coupled with rapid metabolism and elimination, resulting in limited systemic bioavailability [42,43,44,45,46]. In this vein, we developed a polymer-based curcumin formulation (OC-S) that improved the solubility of curcumin by >2000 times and precisely delivered soluble curcumin to the lumen of the colon at the inflamed site and has significantly improved anti-inflammatory and anti-neoplastic efficacy compared with curcumin. Furthermore, we investigated OC-S for its compatibility with dietary products, stability within these products, and the biological activity of the curcumin extracted from them as a proof of concept.

To overcome the limitation of poor systemic bioavailability of curcumin due to extremely low water solubility, poor water stability, and rapid metabolism, we formulated the highly water-soluble OC-S complexes that are delivered locally to the lumen of the colon, the target site for UC and CRC. OC-S was prepared using non-toxic, hydrophilic anionic Eudragit^®^ S100 polymer and curcumin. Our previous report showed that OC-S is superior in aqueous solubility and availability at the local colon site without systemic absorption compared with curcumin alone using healthy mice [24]. However, in patients with colitis, the permeability of the intestine is altered [47,48]. Since colitis-associated diarrhea could pose a challenge to the effective retention of anti-IBD drugs in the colon, we adopted a strategy of charge-based interaction to effectively retain the OC-S in the colon of IBD patients even with diarrhea [29,30,31]. Notably, inflammation of the colonic mucosa is accompanied by depletion of the negatively charged mucus layer and the accumulation of positively charged proteins at the epithelial surface, such as transferrin [30,49], and anti-microbial peptides [50,51,52]. Therefore, OC-S was intentionally designed to carry a negative charge at the pH of the colon with a selection of an anionic polymer (Figure 1A). By using ex vivo colon tissue from colitis mice, it was established that OC-S is anchored to the inflamed colon tissue (Figure 1B,C), potentially through charge-based interactions. Such charge-based interactions improved the uptake and retention of drugs, as shown by other inflammation-targeted delivery systems in IBD [29,30,31]. In this study, we confirmed that when delivered orally, no detectable levels of curcumin were observed in the blood of colitis mice.

Previously, we showed the beneficial effects of OC-S on colonic health in mice subjected to experimental colitis [24]. However, in the current report, we further titrated the lowest dose of OC-S (equivalent to curcumin 5 mg/kg/day) for significant improvements in mice subjected to DSS colitis compared with curcumin. These findings agree with the outcome of our previous study and indicate that OC-S may be a more potent therapeutic agent for attenuating colitis-induced symptoms even at a very low dose [24]. As shown in this study, OC-S was able to provide comparable protection from colitis even at doses 3–60 times lower than previously reported curcumin studies (50–300 mg/kg/day) [53,54,55]. When calculated based on body surface area (1.8 m^2^ for humans weighing ~70 kg), the 15 mg/kg mouse dose (45 mg/m^2^) is equivalent to 81 mg/day for humans [56], which is much lower than the 8–12 g/day dose administered to humans apparently without adverse effects [13,57]. Additionally, OC-S formulation can precisely deliver (dependent on pH) the active curcumin to inflamed and/or injured sites of the colon compared with curcumin, which can affect the entire gastrointestinal tract [24].

Furthermore, in the present study, quantitative RNA expression analysis showed that the anti-inflammatory cytokine IL-10 in the OC-S treatment group was elevated; however, proinflammatory cytokines TNF-α, IL-1, and IL-6 were found to decrease, and IL-10 plays an antagonistic regulatory function in host cell immunity [32,33,34]. Additionally, using LPS, a known intestinal inflammation inducer, we found a similar OC-S effect on IL-6 and IL-10 expression compared with curcumin in intestinal epithelial cells. Several reports suggest that IL-10 can inhibit antigen presentation and downregulate the formation and secretion of IL-1, IL-6, TNF-α, and other critical inflammatory aspects in macrophages and T cells, thus improving IBD intestinal inflammation [32,33,34]. We speculate that OC-S serves an anti-inflammatory role by elevating IL-10 and reducing proinflammatory cytokines, including IL-1, IL-6, and TNF-α. Therefore, recalibrating cytokines levels by OC-S may be a novel strategy in managing IBD disease, including flare-ups and relapses. We previously reported that OC-S acts as an antagonist of TLR4 signaling [24]. Several studies have established that the deregulation of TLR4 and associated signaling promotes IBD severity [58,59,60,61,62], potentially through MyD88 signaling [63]. OC-S may be exerting its anti-inflammatory activities by antagonizing the activation of TLR4 or through its direct effect on NF-kβ.

Extensive research over the past two decades suggests the potential role of curcumin in modulating not only IBD but also CRC development and progression. However, clinical trials on the use of curcumin in chemoprevention in CRC showed mixed results depending on the curcumin dose and treatment duration [64]. Notably, a high dose of curcumin (4 gm/day) induces a 40% reduction in aberrant crypt foci in CRC when delivered systemically [12,65]. On the other hand, patients with mild to moderate IBD treated with 2 to 3 gm/day curcumin uphold IBD reduction clinically and endoscopically devoid of causing harmful effects [35,66,67]. However, studies using high doses of curcumin of up to 12 gm/day have shown adverse side effects like mild gastrointestinal symptoms, such as stomach pain, nausea, and diarrhea in relatively short studies [13]. In another study, daily curcumin intake of 8 gm/day for two months in patients with advanced pancreatic cancers led to abdominal pain ulcers as the predominant side effect [68]. As noted above, recent reports revealed that curcumin supplements may interfere with iron metabolism and should not be taken if you are on blood thinners such as warfarin [69,70,71]. Given that curcumin is needed in high doses due to its low systemic bioavailability, it can elicit detrimental side effects if it is used long-term. In this regard, OC-S formulation showed a greater decrease in cyclin-D1 expression, the upregulation of cleaved caspase-3 expression, and a reduction in tumoroid size compared with equivalent curcumin amount. Further, it is targeted as a local therapy sparing the systemic off-target effects, as well as overcoming the systemic bioavailability challenges. Taken together, the key finding of the current study is that even a low dose of OC-S is enough to treat intestinal inflammation and/or CRC cell growth and is superior to unformulated curcumin [24].

Turmeric dietary supplement use has grown in recent years including in various dietary supplements and has become a top-selling botanical dietary supplement in the United States [72]. However, dietary food sources differ from turmeric supplements or when taken as a pill. The co-mixing of curcumin with dietary products is a convenient approach for patients to mask the taste, improve the ease the administration, and reduce adverse effects in the gut [73,74,75]. However, these dietary supplements are not standardized like prescription medications, meaning the dose is not regulated, compatibility is not investigated, and purity is not guaranteed. Further, the components of the dietary product may interfere with the biological activity of curcumin. In this study, we thoroughly investigated the stability and compatibility of OC-S in various dietary products and further showed that OC-S is functionally active while being a part of these dietary products. These dietary food products include single sachets of pomegranate green tea, mixed fruit drinks, chicken soup, and orange drinks, which can be easily consumed as supplemental products, and their daily consumption could be well regulated. They further represent various hot and cold mixes used in diets. The curcumin was extracted from OC-S-DP sachets at predetermined intervals and analyzed for remaining intact active curcumin in stability evaluation. The OC-S complex is stable in these dietary food supplements for up to 12 months. Additionally, extracted OC-S from these supplements shows curcumin’s functional bioactivity in reducing intestinal epithelial cell proliferation while increasing cell death at a lower concentration than curcumin of intestinal epithelial cells. However, the only limitation of this study is that there is no long-term controlled trial using OC-S supplements in the preclinical setting, which is an ongoing follow-up study in our laboratory. Of note, curcumin food or health booster supplements currently available in the market have comparatively high curcumin doses, and taking them for a long time could be detrimental to human health. A low dose of OC-S in food supplements, which does not absorb into the blood, could provide a better and safer option for long-term use with minimal unwanted side effects.

## 5. Conclusions

In conclusion, this study underscores the pharmacological significance of OC-S as a dietary supplement for long-term use in conjunction with conventional medications for anti-inflammatory and anti-neoplastic purposes. These findings support its potential as a natural remedy for managing inflammatory bowel disease (IBD) and preventing colorectal cancer (CRC).

## Data Availability

The data presented in this study are available in this article and Supplementary Materials.

## Supplementary Materials

The following supporting information can be downloaded at https://www.mdpi.com/article/10.3390/cells13110957/s1: Table S1: Antibodies source information; Table S2: Realtime primers sequences.
