# Supplementary material for: Stable Dietary Ora-Curcumin Formulation Protects from Experimental Colitis and Colorectal Cancer
Source: Cells. 2024 Jun 1;13(11):957. doi: 10.3390/cells13110957 (PMC11172195; doi:10.3390/cells13110957)
Supplement: Supplementary file 1 [file cells-13-00957-s001.zip › cells-2994333-supplementary.pdf]

Supplementary Table S1. Antibodies source information.

	Antibody	Company	Catalog number
1	Cyclin D1	Cell Signaling Technology, Beverly, MA, U.S.A.	2978S
2	Cleaved Caspase-3	Cell Signaling Technology, Beverly, MA, U.S.A.	9661S
3	β-Actin	Millipore Sigma 400 Summit Drive Burlington, MA 01803 United States	A2228
4	Anti-mouse IgG-HRP	R&D Systems, Inc. 614 McKinley Minneapolis, MN 55413	HAF007
5	Anti-Rabbit IgG-HRP	R&D Systems, Inc. 614 McKinley Minneapolis, MN 55413	HAF008

Supplementary Table S2. Realtime primers sequences.

Primer	Sequence
Mouse	
TNF-α Forward	CTGTGAAGGGAATGGGTGTT
TNF-α Reverse	GGTCACTGTCCCAGCATCTT
IL-1 Forward	CTCCATGAGCTTTGTACAAGG
IL-1 Reverse	TGCTGATGTACCAGTTGGGG
IL6 Forward	AGAGACTTCCATCCAGTTGC
IL6 Reverse	TCCTTAGCCACTCCTTCTGT
IL10 Forward	TAGAGCTGCGGACTGCCTTC
IL10 Reverse	CTTCACCTGCTCCACTGCCT
Human	
IL6 Forward	AGACAGCCACTCACCTCTTCAG
IL6 Reverse	TTCTGCCAGTGCCTCTTTGCTG
IL10 Forward	GCC TAA CAT GCT TCG AGA TC
IL10 Reverse	TGATGTCTGGGTCTTGGTTC
TGF-β Forward	CCCAGCATCTGCAAAGCTC
TGF-β Reverse	GTCAATGTACAGCTGCCGCA
